# Shadow of a Shadow: Ferrocyanide and Nitroprusside as Sunscreens for Photosensitive Prebiotic Molecules

**DOI:** 10.3390/life16050856

**Published:** 2026-05-21

**Authors:** Lukas Rossmanith, Sofia K. Platymesi, Samantha J. Thompson, Paul B. Rimmer

**Affiliations:** 1 Cavendish Laboratory, University of Cambridge, JJ Thomson Ave., Cambridge CB3 0HE, UK; sjt20@cam.ac.uk (S.J.T.); pbr27@cam.ac.uk (P.B.R.); 2 Department of Physics, University of Oxford, Denys Wilkinson Building, Keble Road, Oxford OX1 3RH, UK; sofia.platymesi@physics.ox.ac.uk

**Keywords:** UV–Vis spectra, prebiotic chemistry, origin of life, photochemistry, sunscreen, nitroprusside, ferrocyanide

## Abstract

Stellar irradiation is thought to be a significant contributor to the origin of life. Ultraviolet (UV) light interacting with iron cyanide complexes may play an important role in prebiotic chemistry. The UV–Visible (UV–Vis) spectra of these iron cyanide complexes can be measured by the same source that drives the chemistry, providing a real-time in situ quantitative analysis of prebiotically relevant, UV-driven photochemistry. We measure the UV–Vis absorbances of ferrocyanide and nitroprusside, and relate these absorbances to known concentrations. We show that these absorbances can be combined to accurately predict the concentrations of ferrocyanide–nitroprusside mixtures that could be generated from ferrocyanide and nitroxyl salts irradiated by ultraviolet light. The ferrocyanide molar attenuation coefficients were found to be maximal at the following: $\varepsilon_{ferrocyanide}\left(340\;\mathrm{nm}\right)=\left(2.2\pm 0.4\right)\times 10^{3}\;{\mathrm{dm}}^{2}\;{\mathrm{mol}}^{-1}$. Nitroprusside peaks show the following values: $\varepsilon_{nitroprusside}\left(340\;\mathrm{nm}\right)=\left(4.1\pm 0.3\right)\times 10^{2}\;{\mathrm{dm}}^{2}\;{\mathrm{mol}}^{-1}$, $\varepsilon_{nitroprusside}\left(400\;\mathrm{nm}\right)=\left(1.71\pm 0.05\right)\times 10^{2}\;{\mathrm{dm}}^{2}\;{\mathrm{mol}}^{-1}$, and $\varepsilon_{nitroprusside}\left(500\;\mathrm{nm}\right)=62.1\pm 1.7\;{\mathrm{dm}}^{2}\;{\mathrm{mol}}^{-1}$. With the help of our measured absorbances, we consider ferrocyanide and nitroprusside to function as sunscreens. In the absence of continuous ferrocyanide sources, UV-sensitive compounds could be protected on timescales of months. This would allow for compounds like nicotinamide adenine dinucleotide, NADH, to survive for over a year at depths of 5 m, compared to a lifetime of 6 months when unprotected. Our toy model constrains the photochemical survival of compounds of interest to the origin of life community across a comprehensive spectral range and can be used to constrain the survival using different exoplanetary irradiative conditions; thus, we are able to explore the UV environment with the presence of ferrocyanide and nitroprusside and contribute to the wider discussion surrounding the prevalence of the origin of life in the Universe.

## 1. Introduction

Photochemistry is a vital component of prebiotic chemistry. Photochemistry in a natural environment relies on the host star, meaning that a significant part of the chemistry of a planet could depend on the irradiative properties of the host star. Rimmer et al. discuss that generally a hotter star would lead to more photochemistry and higher reaction rates [1]. Hotter stars emit more photons at shorter wavelengths, given that stars are considered to behave like black bodies. This includes higher rates of ultraviolet emission which drives the rate of photochemistry, as higher energy photons are required to trigger a photochemical reaction. Many other reactions judged to be prebiotically relevant are photochemically driven and broadly fit in the cyanosulphidic framework for the origin of life [1,2,3,4,5,6,7,8,9,10,11]. Powner et al. and Ritson et al. are of note because the former showed the possible abiotic synthesis of pyrimidine ribonucleotides and the latter showed the synthesis of basic 2 or 3 carbon sugars [6,7]. Xu et al. proposed a network, driven by photochemistry, to reduce HCN and construct simple sugars as well as precursors of hydroxy acids and amino acids [10].

However, UV light presents its own issues to biochemical compounds and life. Ranjan et al. note that nucleotide photolysis is likely to occur at around 260 nm, although photolysis of individual nucleotide bases occurs at very low quantum yield, generally at values of $10^{-4}$ [12,13,14,15,16,17]. Nevertheless, polynucleotide strands, like RNA strands, are more vulnerable to UV degradation at 260 nm; Kladwang et al. found that it only took 600 s to cause significant damage to RNA strands with UV at an exposure rate of approximately 0.2–0.8 mJ cm^−2^ s^−1^ [18]. Furthermore, it is widely known that UV irradiation can cause damage to RNA and DNA strands. One common type of damage that DNA strands can sustain are cyclobutane pyrimidine dimer (CPD) lesions, which occur when two adjacent pyrimidines undergo photochemical cycloaddition to form a cyclobutane bridging ring [19]. If the dimer is formed from two thymine bases and guanine and adenine are adjacent, then such dimers can be repaired under further UV irradiation [20].

Previous findings show that canonical nucleobases are more resistant to UV irradiation than non-canonical bases; this suggests that UV irradiation played a role in the natural selection of the canonical bases as carriers of genetic information [21]. Nevertheless, Bucher et al. required particular nucleotide chains [20]. Therefore, canonical bases may be more resistant, but are still prone to UV degradation. Here degradation refers to any process by which a compound’s original function and structure was altered through a photochemical reaction. This means that not all UV damage can be repaired and sustained UV irradiation will impair the functions of nucleotide chains. Indeed, photodamage to DNA chains is a known problem for the origins of life but also for the modern biosphere, with photodimerization being a common lesion caused by UV irradiation and corrupting the genetic information present in the chain [22,23,24]. UV damage is not limited to nucleotide chains. UV irradiation was found to alter lipid and protein conformations, causing functional and structural damage to these vital biomolecules [25]. Such effects were observed across the entire UV spectrum. Adenosine triphosphate (ATP) and nicotinamide adenine dinucleotide (NADH), which are key compounds in cellular metabolism, are both susceptible to UV irradiation in different regions of the UV spectrum [26,27]. ATP is susceptible to 200–300 nm irradiation whilst NADH is susceptible to 300–400 nm irradiation. UV has therefore been seen as one of the most effective ways to sterilise space probes, as UV kills micro-organisms whilst avoiding damage to any vital machine component [28]. As prebiotic systems approach higher levels of complexity, i.e., nearing the complexity of biochemical systems via the production of nucleotide chains, lipids and proteins, they become more susceptible to UV degradation.

Prebiotic environments rarely present themselves as environments with uniform conditions that are constant with time. Gradients of various types are important to consider when evaluating any prebiotic scenario. Wet–dry cycling, which creates a time-dependent gradient in the water level of lakes like Goodenough and Last Chance, is thought to have enabled concentrations of phosphate as high as 37 mM: a key ingredient needed for the phosphorylation of nucleosides [29]. Furthermore, work on closed basin lakes by Walton et al. supports their ability to concentrate phosphorous compounds [30]. Consequently, it is thought that wet–dry cycling allowed for prebiotic lakes to build up the necessary feedstocks at sufficient concentrations to drive prebiotic chemistry forward. Thermal gradients have also been considered as important contributors to prebiotic environments. Hydrothermal vents provide flow, due to the temperature difference across the vent, allowing for prebiotic synthesis to occur [31,32]. However, such hydrothermal vents typically are present at ocean depths of several thousand meters where no UV flux can reach due to scattering and absorption across large path lengths [17,31]. This would result in no photochemistry being possible. Deep sea vent scenarios lie outside the class of scenarios considered in this paper.

Here we propose that a different depth-dependent UV gradient could have been necessary for the development of biochemical compounds from prebiotic environments. If prebiotic environments allowed for both UV-induced photochemistry whilst also providing a refuge for more complex compounds, then the cyanosulphidic network could proceed and compounds such as nucleobases, lipids, and amino acids could be protected. Strongly absorbing UV compounds could be useful in this regard as they would be able to act as sunscreens, shielding susceptible compounds from irradiation. Compounds, such as ferrocyanide, absorb UV light and use its energy to undergo photochemical reactions in solution [11,33]. The surface of a water body (e.g., lake), exposed to irradiation, would still be host to the expected photochemistry; however, due to the presence of the sunscreens, lower layers would be shielded, creating a UV intensity gradient along the water column.

Ferrocyanide and nitroprusside have been proposed to be key prebiotic molecules. Todd et al. provides experimental support for how ferrocyanide can concentrate in alkaline lakes at a pH of 8 [34]. Toner et al. discusses the possibility of carbonate alkaline lakes on early Earth [35]. It is plausible such lakes existed, as ferrocyanide under these conditions can efficiently concentrate cyanide from rainout and iron from Fe(II) weathering into the transition metal complex [34]. Ferrocyanide is sensitive to UV light [33]. Photo-oxidising ferrocyanide releases solvated electrons that are useful in prebiotic chemistry, as they create the energetic conditions required for further chemical reactions [10]. Photo-aquating ferrocyanide produces hydrogen cyanide, that can participate in a variety of prebiotic syntheses [36]. The photo-oxidised product, ferricyanide, can be recycled into ferrocyanide via reduction by sulphite [10]. Xu et al. showed that ferrocyanide could accelerate photochemical processes as it is capable of acting as a catalyst [10].

In order to form nucleotide chains non-enzymatically, Phosphorylated nucleotide bases need to be activated. Phosphorimidazolides, such as ImpA, can be used in the non-enzymatic oligomerisation (i.e., limited polymerisation) of phosphorylated nucleotides [37,38,39,40]. Methyl isocyanide can activate a phosphorylated nucleotide base, adenosine 5′-monophosphate (AMP), to eventually form the phosphorimidazolide of AMP [9,41]. Via methyl isocyanide, the building blocks of RNA and amino acids can be produced [42]. Mariani et al. showed how methyl isocyanide could be formed from nitroprusside, whilst nitroprusside itself can be formed from ferrocyanide in the presence of UV light and nitrite (${\mathrm{NO}}_{2}^{-}$) or nitrate (${\mathrm{NO}}_{3}^{-}$) [9,11]. For these reasons, ferrocyanide and nitroprusside are important prebiotic molecules because they feed into networks that are thought to be important in the formation of more complex biomolecules.

Ferrocyanide and nitroprusside are strongly absorbing in the UV region [17,33,43,44]. Alkaline lakes could be home to significant concentrations of both [17]. Therefore, ferrocyanide and nitroprusside could act as good ‘sunscreens’, providing a depth-dependent UV gradient. The ability of ferrocyanide to absorb UV and act as a sunscreen has been experimentally investigated before; however, such studies focus on irradiation under 300 nm, which is of interest since compounds like glycine and polynucleotides degrade in this region [17,44,45]. Yet ferrocyanide and nitroprusside could absorb in regions above 300 nm and so protect compounds, like NADH, that are susceptible to this irradiation [27,33,43]. Therefore, it is important to understand how well ferrocyanide acts as a sunscreen in the 300–400 nm region. Furthermore, a comprehensive study of the molar absorption coefficients of ferrocyanide and nitroprusside across both the UV and visible region has not yet been performed, nor has the effectiveness of nitroprusside acting as a sunscreen been investigated (favouring investigations of ferrocyanide only). The effectiveness of shielding would be dependent on the survival of ferrocyanide and nitroprusside as well as the production fluxes of both. Should shielding be effective and sustainable, then the downstream products of prebiotic photochemistry could develop deeper in the lake without UV degradation [46]. A post-impact climate provides long timescale of HCN rainout with the presence of a titan-like haze [47]. Large possible Fe(II) fluxes point to iron rich lakes being plausible [33,35]. Under the protection of the haze, a large concentration of ferrocyanide could build up. As the haze clears, the subsurface layers of the lake could then already be protected by ferrocyanide.

**Figure 1:**
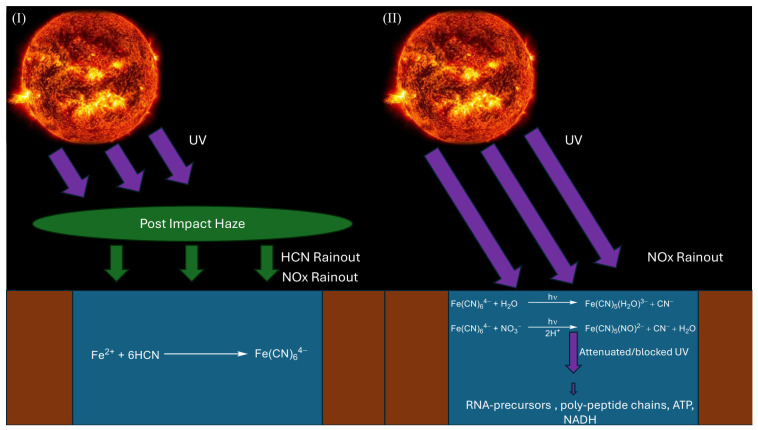
A stagnant lake in a post-impact environment [47]. (**I**) Initially, there is an organic haze that blocks UV flux from reaching the lake, allowing ferrocyanide to build up from both Fe^2+^ sources and HCN rainout from the haze [33,47,48]. (**II**) Once the haze clears, UV flux, from the Sun, irradiates the lake and triggers photochemical reactions involving ferrocyanide and nitroprusside. Absorptions by both complexes attenuate the UV flux that reaches the deeper parts of the lake and thus lowers the rates of the photochemical reactions. This protects photosensitive compounds which reside in lower depths of the lake. In both (**I**) and (**II**) NOx rainout, from lightning reactions and vital for the supply of nitrate to form nitroprusside, is assumed to remain constant.

We investigate this scenario by building a toy lake model. We consider a stagnant lake in a post-impact environment where initially an organic haze blocks UV irradiation and allows for ferrocyanide to build up [47,48]. Since closed basin lakes have no inner flow patterns, geological features such as hydrothermal vents would not distribute material through the water column and are therefore not considered in this model [30]. Once the haze clears, the lake is exposed to UV irradiation and ferrocyanide either photo-aquates or photo-oxidises in the presence of nitrate to nitroprusside (Figure 1) [11,33]. With ferrocyanide and nitroprusside absorbing UV flux, they could protect other sensitive compounds, deeper in the lake, as long as they are present in sufficient concentrations. We model the concentrations of ferrocyanide and nitroprusside at different depths and times, using rate constants from Rimmer et al. and Todd et al. [11,33]. We also include the lake geometries from Todd et al. [34]. We aim to estimate an upper limit of ferrocyanide survival, using favourable conditions from Todd et al. [33]. Using this, we calculated how the irradiation flux changes with time at different depths, to constrain how well ferrocyanide and nitroprusside can act as sunscreens within the lake. We use FlareLab, a variable-UV reactor and UV–Vis spectrometer, to measure the absorbances of ferrocyanide. We use the measurement values to calculate their molar attenuation coefficients, which we use in our model to probe the degree of UV attenuation ferrocyanide and nitroprusside at different lake depths. This model works within the confines of the cyanosulphidic scenario, as photochemistry plays a central role in it [6,46]. However, our model would be relevant to any other scenarios that have the requisite conditions, i.e., a stagnant lake and photochemistry as the main driving force behind chemical reactions. Consequently, our model need not be limited to origin of life scenarios; yet, for the purposes of this paper, we centre the discussion around prebiotic chemistry, UV environments and how they could impact abiogenesis. We recognise that other scenarios could have been possible, but for this work, we shall not consider them.

In Section 2, we detail the setup of how we produce our absorbance spectra and how our lake model is constructed. In Section 3, we show results for nitroprusside and ferrocyanide concentrations, how they relate to their UV attenuation, and predict the local UV environment for iron–cyanide-rich lakes. We discuss the implications of these results in Section 4. Section 5 contains our conclusions.

## 2. Methods

In this section, we detail the experimental methods and calculations employed in our work with FlareLab. In Section 2.1, we detail the set up of FlareLab used to measure our standards and show its irradiative properties. In Section 2.2, we show how we make our standard solutions of nitroprusside and ferrocyanide, as well as which experimental mixtures of both. In Section 2.3, we detail how we compute our data into UV–Vis absorption spectra and recover molar extinction coefficients for ferrocyanide and nitroprusside via linear regression. Finally, in Section 2.4, we detail how we build our lake model and how our experimental results feed into it.

### 2.1. FlareLab

**Figure 2:**
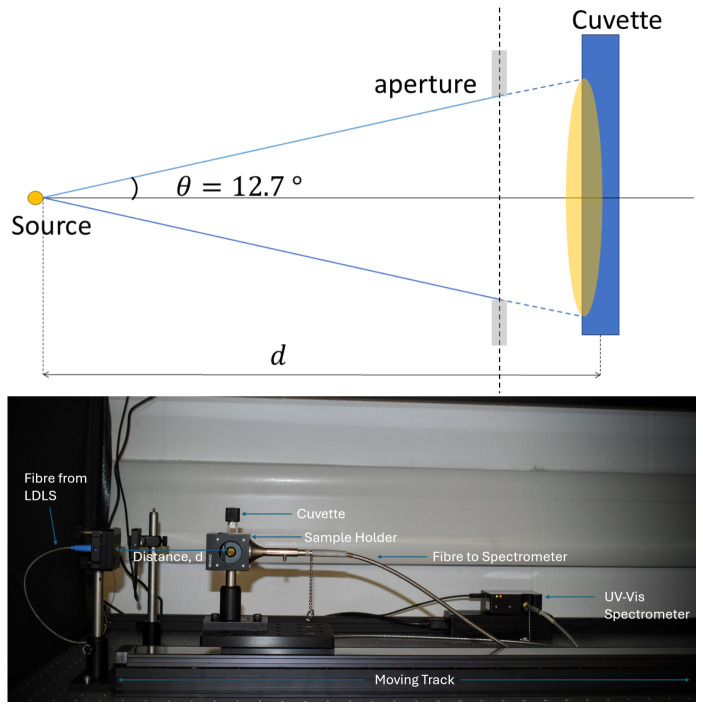
Schematic (**top**) and photo (**bottom**) of FlareLab. The light source is an LDLS by Energetiq (model EQ-99X-FC-S)—the light output is diverging with a cone angle of 12.7° due to the numerical aperture of the fibre. The cuvette holder is placed on a motorised linear stage, allowing a variation in the incoming intensity by varying the distance (d) from the source. A fibre optic cable is attached to the cuvette holder, leading to a FLAME-S-UV-Vis UV–Vis spectrometer, which is visualised with OceanView software.

**Figure 3:**
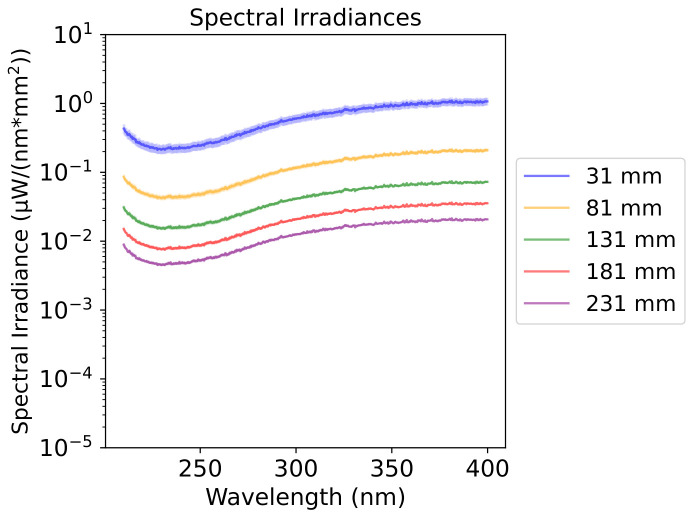

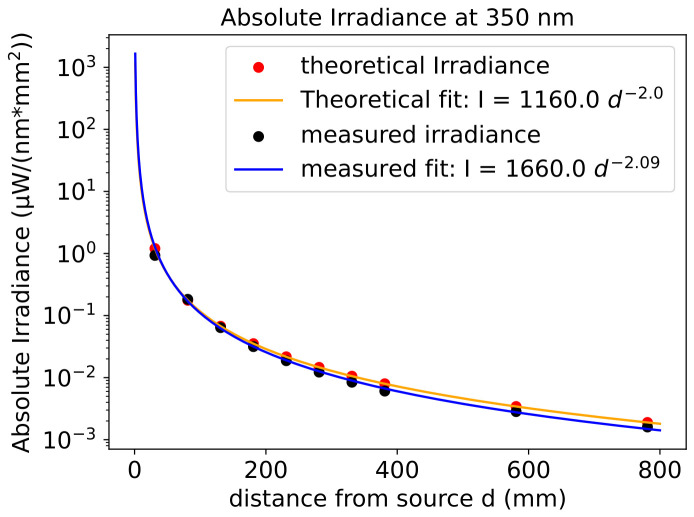
Received spectral irradiance of our light source with distance dependence. (**Top**) Spectral irradiance profile of the Laser-Driven Light Source (LDLS), in units of μ${\mathrm{Wnm}}^{-1}{\mathrm{mm}}^{-2}$ as function of wavelength, with the detector at different distances from the emitter. (**Bottom**) Spectral irradiance of the LDLS, in units of μ${\mathrm{Wnm}}^{-1}{\mathrm{mm}}^{-2}$ at 350 nm, showing its expected $d^{-2}$ dependence, with d being the distance between the source and the sample in units of mm.

FlareLab is the experimental setup used in this work, which broadly consists of a fibre-fed light source and a set of opto-mechanical components placed in an enclosure (Figure 2). A motorised linear stage on a 750 mm length track is used, upon which a cuvette holder is mounted. The linear stage used in FlareLab is a Zaber X-LRT-AEC Series model (Firmware: 7.22.10079)—this has a built-in linear encoder and motor controller which, via computer control, allows precise positioning and motion control. A fixed, fibre-fed light source is located at one end of the track. The optical UVFIBERX-455 fibre (Wilmington, MA, USA, Energitiq) used for the output of the Energetiq EQ-99X-FC-S model (Wilmington, MA, USA, Energitiq) Laser-Driven Light Source (LDLS) has a numerical aperture of 0.22, which sets the cone angle of the emitted light as shown in Figure 2. This allows for incoming UV intensity to be varied with distance (Figure 3). This is a broadband (190–2500 nm) white light source with a reasonably flat spectral profile, though the optical fibre choice here also limits the bandwidth of the emitted light—in this case, to approximately 190–900 nm. Prepared samples in cuvettes are placed in the cuvette holder and irradiated from a specified distance. A fibre-fed spectrometer is connected to the back side of the cuvette holder. We used a UV–Vis FLAME-S-UV-Vis Spectrometer from Ocean Optics (Ostfildern, Germany) to measure the absorbances of the samples. The spectrometer fibre entrance also included a cosine corrector to maximise light collection. The FLAME-S-UV-Vis (Ocean Optics, Ostfildern, Germany) has a wavelength range of 200–800 nm; the data is exported via Ocean Optics’ OceanView software (v1.6.7) and plotted using a Python script (Python 3.10.12).

By measuring absorbances of different nitroprusside and ferrocyanide concentration at different distances in triplicate, we lower our error in absorbance measurements and increase the quality of the molar extinction values used in our lake model. FlareLab components have previously been used to successfully measure rhomboclase absorbance [49].

### 2.2. Making and Measuring Standards

We make nitroprusside and ferrocyanide standards to measure their concentration dependent absorbance using FlareLab.

To measure nitroprusside absorbance, we add sodium nitroprusside dihydrate (Na_2_[Fe(CN)_5_(NO)]·2H_2_O, 297.95 g/mol, 5.959 g) to argon-degassed distilled water (20 mL) to make an initially concentrated 1 M solution. 10 μL of the 1 M solution is added to argon-degassed distilled water (9900 μL) to form a 0.001 M nitroprusside solution. Dilution of the 1 M and 0.001 M solutions achieve the concentrations of $10^{-6}$ M–1 M of nitroprusside (Table A1). This process is repeated for making ferrocyanide solutions. To measure ferrocyanide absorbance, we add sodium ferrocyanide dehydrate (Na_4_[Fe(CN)_6_]·10H_2_O, 484.06 g/mol, 0.968 g) to argon-degassed distilled water (20 mL) to make an initially concentrated 0.1 M solution. 100 μL of the 0.1 M solution is added to argon-degassed distilled water (9900 μL) to form a lesser concentrated 0.001 M ferrocyanide solution. Dilution of the 0.1 M and 0.001 M solutions achieve concentrations of $10^{-6}$ M–0.1 M of ferrocyanide via further dilution (Table A2).

All concentrations of ferrocyanide and nitroprusside are measured in triplicate and at a range of distances across the track to verify that we obtain the same absorption features for all distances of FlareLab. The distances chosen are 81 mm, 231 mm, and 581 mm from the source. Using FLAME-S-UV-Vis and OceanView, the UV–Vis spectra of each run are recorded and processed.

Another part of the experiment tests whether the mathematical combination of the standards can match up to the experimental combinations of the standards, to verify the absorbances given by FlareLab. A series of experimental mixed standards are made and also tested in triplicate and at the same distances as before (Table A3). To dilute the nitroprusside to the desired concentration, a 1 M solution is used as before. In some cases, sodium nitroprusside dihydrate (${\mathrm{Na}}_{4}\left[\mathrm{Fe}{\left(\mathrm{CN}\right)}_{5}\left(\mathrm{NO}\right)\right]$·$2H_{2}O,\;297.95\;g/\mathrm{mol}$) is weighed and directly added to the solution to obtain the desired concentration. For ferrocyanide, a 0.1 M solution is used to dilute to the correct concentration. As with the single component solutions before, the mixed, aqueous solutions have a total volume of 2 mL.

### 2.3. Retrieving Absorbance Data

To make our absorption spectra, we measure the electron counts registered by our UV–Vis spectrometer, resulting from photon absorption per wavelength onto the device. The exposure time is set to take full advantage of the dynamic range of the detector, to obtain the best signal-to-noise ratio. The background and initial reference counts are measured at the start of each set of runs for each distance (Section 2.2). The background was obtained by measuring counts per wavelength with the source turned off. The initial reference is obtained by directing light through a cuvette filled with water. This is to remove the effect of photon scattering caused by the water. Photon scattering could potentially create false absorption readings or skew the measurement by deflecting more light towards the detector. Finally, the photon counts with light shining through the samples are measured. The three elements are then combined to give a relative absorption shown in Equation (1):

(1) $$r\left(\lambda \right)=1-\frac{n_{m}\left(\lambda \right)-n_{b}\left(\lambda \right)}{n_{ref}\left(\lambda \right)-n_{b}\left(\lambda \right)}=1-\frac{I}{I_{0}}=1-e^{-\tau (\lambda )},\;$$

where r(λ) is the relative absorbance per wavelength, $n_{m}\left(\lambda \right)$ are the photon counts measured per wavelength, $n_{b}\left(\lambda \right)$ are the photon counts from the background per wavelength, and $n_{ref}\left(\lambda \right)$ are the photon counts with the reference cuvette per wavelength. $I/I_{0}$ is the ratio of the incoming intensity, $I_{0}$, and the intensity that makes it through the measured sample, *I*. Finally, τ(λ) is the optical depth.

We take this approach to visualise explicitly how many photons from our light source are absorbed. This will show in which regions the absorbance signal merges with the noise of spectrometer. Equation (1) relates to the standard decadal absorbance via $log\left(I/I_{0}\right)=-\varepsilon \left(\lambda \right)c\beta$, where β is the path length, *c* is the concentration, and ε(λ) is the wavelength-dependent molar extinction coefficient.

Using r(λ) and the corresponding wavelengths, the UV–Vis spectra of the various standards are plotted and the triplicate runs are compared. This method is used to make the spectra of the solutions that are measured directly using FlareLab. This includes the solutions containing only ferrocyanide or nitroprusside as well as the solutions containing a mixture of both.

To simply estimate the errors that arise from a complex mix of the instrument set up, inherent fluctuations in the light source, weighing, diluting, and mixing errors, we take the standard deviation. Each spectrum of standard is measured across three distances each three times, which gives a standard deviation across 9 data points.

#### 2.3.1. Mathematically Combining Standard Absorption Features

We take the measured individual spectra for nitroprusside and ferrocyanide and mathematically combine them to compare them with the mixed standards (described in Section 2.2) and to compare ferrocyanide absorption features with nitroprusside features.

We consider the following equation:

(2) $$\tau \left(\lambda \right)=\sum_{i=1}^{N}\sigma_{i}\left(\lambda \right)n_{i}\beta ,\;$$

where β is the path length of the cuvette (0.1 dm) and *N* signifies the number of absorbers in solution; *N* = 2 when ferrocyanide and nitroprusside are present together and *N* = 1 when it is either ferrocyanide or nitroprusside; $\sigma_{i}\left(\lambda \right)$ is the attenuation cross-section of the species contributing to the absorption (in units of ${\mathrm{dm}}^{2}$) and $n_{i}$ signifies the number density of the species (in units of ${\mathrm{dm}}^{-3}$). It is important to note that $\sigma_{i}\left(\lambda \right)$ depends on wavelength and that even in a mixture, there exist wavelength regions where one species has negligible absorbance; therefore, its cross-section is effectively zero and thus does not contribute to the shape of the end spectrum at that wavelength. $c_{i}=n_{i}/N_{A}$ and $\varepsilon_{i}\left(\lambda \right)=N_{A}{\left(\ln\left(10\right)\sigma_{i}\left(\lambda \right)\right)}^{-1}$ (concentrations of the species and molar attenuation coefficient, respectively). The following equations convert from $n_{i}$ and $\sigma_{i}\left(\lambda \right)$ ($N_{A}=6.022\times 10^{23}\;{\mathrm{mol}}^{-1}$ is Avogadro’s number).

We use

(3) $$\sigma_{i}\left(\lambda \right)=-\frac{\ln(1-r(\lambda ))}{\beta N_{A}c_{i}},\;$$

where r(λ) is the relative absorbance, β the path length, $N_{A}$ is Avogadro’s number, and $c_{i}$ the concentration of a species. We use this to calculate $\sigma_{i}\left(\lambda \right)$ from the single standard runs of nitroprusside and ferrocyanide. The results feed into Equation (2), giving a total optical depth per wavelength for a solution combining given concentrations of nitroprusside and ferrocyanide. The new total τ(λ) then feeds into Equation (1), producing a new mathematically combined spectrum of nitroprusside and ferrocyanide at given concentrations.

We propagate our error on the premise that the total absorption cross-section of the mixture is a linear sum of the constituent molecular cross-sections. The error in the total absorption cross-section therefore determines the error in the combined absorption.

#### 2.3.2. Linear Regression

For the spectral peaks of the various spectra we verify if these peaks follow a linear progression. From Equation (3), we deduce that ln(1−r) should be proportional to $c_{i}$: the concentration of the absorbing species. Therefore, the gradient of the linear fit:

(4) $$m=-\beta N_{A}\sigma_{i}\left(\lambda \right),$$

gives us the absorption cross-section of the species at that wavelength and the corresponding molar attenuation coefficient:

(5) $$\varepsilon_{i}\left(\lambda \right)=\frac{-m}{\beta \ln(10)},$$

by converting from $\sigma_{i}$ to the molar attenuation coefficient.

We fit the data points via linear regression using the python package scikit-learn [50]. Using this, we calculate the $R^{2}$ factor and the resulting residuals. Using the above equations and the python package, we assess how well the data fits a Beer–Lambert progression and how accurate our results for the molar attenuation coefficients are for the species. Note that this method works best if either the data comes from a single absorber solution or the peak is in a range where one of the species does not absorb (and so $\sigma_{i}\left(\lambda \right)=0$ for one species and not for the other).

Δm is the standard error in the gradient. We then substitute $\varepsilon_{i}\left(\lambda \right)$ into:

(6) $$\Delta \varepsilon_{i}\left(\lambda \right)=\varepsilon_{i}\left(\lambda \right)\frac{\Delta m}{m},$$

which assumes that the fractional error in the gradient is comparable to the fractional error of the molar attenuation coefficient. We evaluate the accuracy of our molar attenuation coefficients derived from FlareLab. In turn, we can then determine how well FlareLab functions as a quantitative measurement system.

### 2.4. Lake Model

Moving to the modelling component of this work, we explore the possibility of ferrocyanide and nitroprusside acting as sunscreens by constructing a lake toy model. We consider two lakes with depths of d = 1 m or d = 5 m; both have a surface area of $\sigma_{lake}=10^{3}\;m^{2}$ and a volume of $V_{lake}=10^{3}\;m^{3}$ or $V_{lake}=5\times 10^{3}\;m^{3}$, respectively; they have a common catchment factor of C = 500, which is analogous to the input variables of the lake model used in Todd et al. [34]. Todd et al. showed shelf-similarity in their ferrocyanide production results with respect to the catchment factor of the lake [34]. We therefore expect that production will scale with C. We assume the lake is stagnant throughout and divide the lake in *n* = 100 layers, such that each layer is 1 cm thick (Figure 1). At each layer, we model the time-dependent abundances of ferrocyanide and nitroprusside after the post-impact haze clears (as described by Wogan et al.) [47]. In addition, we take into account the concentration of ferrocyanide and nitroprusside has on the UV–Vis flux at various depths of the lake.

#### 2.4.1. Post-Impact Haze

Our lake exits within a post-impact climate which follows a progression modelled by Wogan et al.; therefore, it will capture HCN from rainout at a rate of $10^{8}\;\mathrm{molecules}\;{\mathrm{cm}}^{-2}\;s^{-1}$ for $10^{3}$ years, during which a titan-like haze is assumed to exist in the atmosphere [47]. Owing to the low albedo titan hazes have in the UV region, we assume that no UV light reaches the lake surface whilst the haze exists [48]. As HCN rains into the lake, it will react with the Fe(II) present to form ferrocyanide, but as no UV is present, and thermal degradation is ignored, ferrocyanide will gradually accumulate within the lake whilst nitroprusside is not formed [33,51,52]. Assuming the lake is iron-rich, which is plausible given the Fe(II) flux values used in Toner et al. and Todd et al., we find that concentrations of ferrocyanide could reach values well above its solubility limit [34,35]. As such, we limit the concentration of ferrocyanide in the lake at 0.1 M, assuming that the excess would settle on the lake’s floor.

#### 2.4.2. Haze Clears

**Figure 4:**
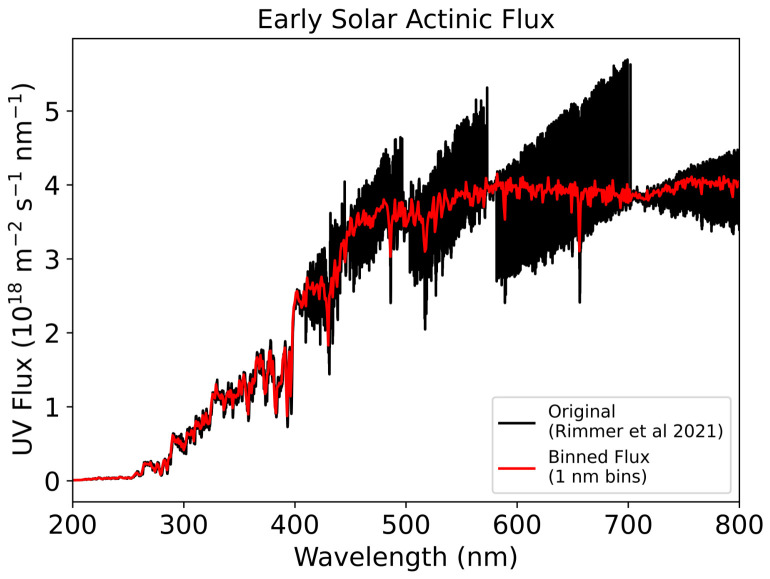
Early solar actinic flux binned to 1 nm (red) from data used in Rimmer et al. (black), which is binned to 1 Å [11]. Our 1 nm bins allow for consistency with our other input data, such as molecular attenuation coefficients, in our lake model.

Once the haze clears, UV light can reach the surface of the lake and HCN rainout ends. During this time, we consider no extra ferrocyanide flux, whilst photo-aquation of ferrocyanide and the production of nitroprusside is possible. Various past studies have explored the formation of nitroprusside from ferrocyanide from photo-oxidation, and have constrained its rate of formation given early sun irradiation [9,11,53,54]. We assume that the nitroprusside photo-aquates at the same rate as ferrocyanide. In addition, we consider nitrate flux due to lightning reactions, where we assume that all NOx produced by lightning (within the catchment area) eventually transforms to nitrate within the aqueous phase of the lake to react with ferrocyanide to nitroprusside in the presence of UV light. Our lightning rate, $l_{r}$, is set at $6.5\;\times \;10^{-5}\;{\mathrm{Jm}}^{-2}\;s^{-1}$ [55]. The ratio of NOx lightning production, $r_{{\mathrm{NO}}_{x}}$, is set at $2\;\times \;10^{-9}\;J^{-1}\;\mathrm{mol}$ [56]. We solve the following ODEs to predict the time-dependent abundances of ferrocyanide ([A]), nitroprusside ([B]), and nitrate ([${\mathrm{NO}}_{3}^{-}$]):

(7) $$\begin{array}{cc} \frac{d[A]}{dt} & =-k_{1}\left[A\right]\left[{\mathrm{NO}}_{3}^{-}\right]-k_{2}\left[A\right],\\ \frac{d[B]}{dt} & =k_{1}\left[A\right]\left[{\mathrm{NO}}_{3}^{-}\right]-k_{2}\left[B\right],\\ \frac{d[{\mathrm{NO}}_{3}^{-}]}{dt} & =C\frac{l_{r}r_{{\mathrm{NO}}_{x}}\sigma_{lake}}{V_{lake}}-k_{1}\left[A\right]\left[{\mathrm{NO}}_{3}^{-}\right], \end{array}\;$$

where $k_{1}=2.59\;\times \;10^{-7}\;s^{-1}$ is the rate constant of the photochemical production of nitroprusside derived from Rimmer et al. and $k_{2}=5.13\;\times \;10^{-5}\;s^{-1}$ is the rate constant of the photo-aquation of ferrocyanide derived from Todd et al. [11,33]. Both rate constants apply in an early solar irradiative environment (Figure 4). We aim to estimate an upper limit of ferrocyanide and nitroprusside production; thus, we use the most favourable $k_{2}$ value from Todd et al., which implies a lake at pH = 8–9 at a temperature T = 280 K [33]. Evidence that such cold Hadean environments could have existed has been supplied by Kadoya et al., who state that nearly frozen lakes were possible [57]. For $k_{1}$, we use the value corresponding to the production of nitroprusside with only nitrate. Rimmer et al. states that the timescale to produce nitroprusside with nitrite is lower; however, it is more plausible that larger quantities of nitrate exist under prebiotic reactions [11].

#### 2.4.3. Lake Layers and UV Attenuation

For each layer, we track chemical evolution based on the ODE solutions. As the concentrations of ferrocyanide and nitroprusside change, the UV–Vis flux changes as the amount of photons absorbed at each wavelength changes [1,11]. The rate constants at layer *n* are dependent on how the photon flux changes at layer n−1. Concentrations of nitroprusside and ferrocyanide at layer n−1 will absorb photon flux in the UV and visible regions and thus determine the photon flux at layer *n*. In addition to absorptions due to ferrocyanide and nitroprusside, we also consider the absorption contributions of typical ions found in ferrous lakes as defined by Ranjan et al. [17,58]. We consider these ions (such as Cl^−^, I^−^, ${\mathrm{NO}}_{3}^{-}$, and ${\mathrm{SO}}_{3}^{2-}$) to exist in the lake at uniform concentrations and have taken the upper limit of their ferrous lake concentrations [17]. Combining this with attenuation from ferrocyanide and nitroprusside results in the total photon flux attenuation that applies to layer *n*. Because photochemical rate constants can be scaled with irradiation flux linearly, we can calculate the rate constants of our reactions at each layer.

We track how rate constants change with time by considering how the absorptions of ferrocyanide and nitroprusside change between layer n−1 and *n* and with time. From Equation (2) we can combine the concentrations of nitroprusside and ferrocyanide with their respective molar attenuation coefficients at each time step and wavelength to obtain z(λ,t), which is the ratio of flux between layers n−1 and *n* at time *t* and wavelength λ. Our model uses an even time-step of 0.07 days and wavelength bins 1 nm wide. We iterate through the layers, apply z(λ,t) between all layers, to time-wise modify the spectrum shown Figure 4 at each layer. $\chi^{n}\left(t\right)$ is the time-dependent ratio between the UV flux integral at layer *n* and the lake surface (n=0):

(8) $$\chi^{n}\left(t\right)=\frac{\int F_{UV}^{n}\left(\lambda ,t\right)d\lambda }{\int F_{UV}^{0}\left(\lambda ,t\right)d\lambda },$$

where $F_{UV}^{n}\left(\lambda ,t\right)$ is the UV flux at the n-th layer and $F_{UV}^{0}\left(\lambda ,t\right)$ is the UV flux at the surface. Because these values are time-dependent, we update our rate constants with the equation $k_{1,2}^{n}=\chi^{n}\left(t\right)k_{1,2}^{0}$ at each time-step, where $k_{1,2}^{0}$ is the unattenuated value of $k_{1}$ or $k_{2}$.

By attenuating the rate constants, we can calculate the rate of ferrocyanide degradation and nitroprusside production at every depth of the lake at every time-step. This will show us how long these two compounds survive in the lake.

## 3. Results

In the following, we show our experimental FlareLab results as well as the results from our lake model. We calculate the molar attenuation coefficients of ferrocyanide and nitroprusside via linear regression. These values are fed into the lake model to give the survival of ferrocyanide and nitroprusside. We show that, in the absence of continuous ferrocyanide sources, UV-sensitive compounds could be protected on timescales of months.

In Section 3.1, we show the corrections applied to our raw data. In Section 3.2, we show our measured absorption curves for varying concentrations of nitroprusside and ferrocyanide standards. We also showcase what the mathematical combination of these two curves looks like. In Section 3.3, we focus on particular absorption peaks of ferrocyanide and nitroprusside and use linear regression to determine their respective molar attenuation coefficients. We then compare the mathematically combined absorption curves to the respective experimental mixtures in Section 3.4. All previous results then feed into our lake model and we show its results in Section 3.5.

### 3.1. Impact of the Background

**Figure 5:**
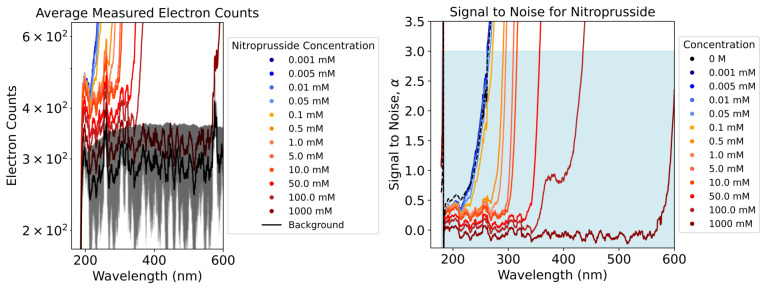
(**Left**) Zoomed-in graph showing the average raw electron counts measured by the UV–Vis FLAME-S-UV-Vis Spectrometer at different nitroprusside concentrations as well as the background. The shaded region represents the error in the background. Features of the background are increasingly visible at higher concentrations where larger blanket absorptions occur. (**Right**) Zoomed-in graph showing the ratio of the count difference (sample count–background count) and background count with varying concentrations of nitroprusside. We exclude measurements in ranges where the ratio is below 3 as a cut-off value.

Features of the background appear in our raw electron counts measured by the UV–Vis FLAME-S-UV-Vis Spectrometer. These are scaled artefacts due to the background and measurement system. We consider the signal-to-noise ratio

(9) $$\alpha =\frac{n_{m}\left(\lambda \right)-n_{b}\left(\lambda \right)}{n_{b}\left(\lambda \right)},\;$$

to remove measurement values that belong to the background. Here, $n_{m}\left(\lambda \right)$ is the measured sample count, $n_{b}\left(\lambda \right)$ is the background count, and α is the ratio between $n_{m}\left(\lambda \right)-n_{b}\left(\lambda \right)$ and $n_{b}\left(\lambda \right)$. This allows us to solely show absorbances due to molecular interaction with the light, rather than absorbance due to different levels of noise. If α<3, then we exclude the sample measurement values within that range as the background was judged to be the dominant factor for the measured features there. Three is the chosen cut-off value for the ratio for the minimum confidence in the presence of a signal, as we can be confident in the validity of the signal. This procedure is applied to both ferrocyanide and nitroprusside results (Figure 5).

As the concentration of either compound increased, we found larger wavelength regions returning blanket absorptions of r(λ)=1. This necessitated larger exclusions of measured data, because for these regions our exclusion criteria of α<3 was met. Larger sections of the spectrum would become noise dominated due to the low levels of detected light.

### 3.2. Absorption Features of Nitroprusside and Ferrocyanide

**Figure 6:**
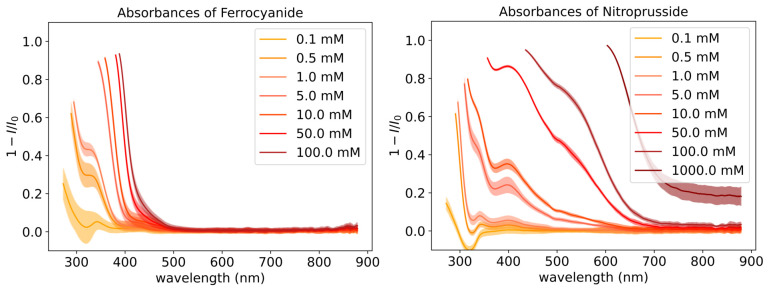
(**Left**) Absorbance of ferrocyanide, ${\left[\mathrm{Fe}{\left(\mathrm{CN}\right)}_{6}\right]}^{4-}$, depending on its concentration. Error region derived from experimental tests in triplicate and from measuring the absorbance of each concentration at varied distances from the emitter. Lower concentrations than 0.1 mM were measured but this yielded only noise and no appreciable peaks with our set up. (**Right**) Absorbance of nitroprusside, ${\left[\mathrm{Fe}{\left(\mathrm{CN}\right)}_{5}\left(\mathrm{NO}\right)\right]}^{2-}$, depending on its concentration. Error region derived in the same way as ferrocyanide. Lower concentrations than 0.5 mM were measured but this yielded only noise and no appreciable peaks with our set up.

Figure 6 shows the UV–Vis absorption features of ferrocyanide and nitroprusside dependent on concentration. Standard tests below 0.1 mM and 0.0005 mM of ferrocyanide and nitroprusside, respectively, did not show good absorption features with FlareLab and so are not shown in these plots. The error bars of the spectra show that it is possible to distinguish the main absorption features between the different concentrations. There is minimal overlap of the error regions, especially at higher concentrations, as it is generally easier to detect absorption features when the abundance is higher. However, these spectra show that FlareLab is also sensitive to lower concentrations.

**Figure 7:**
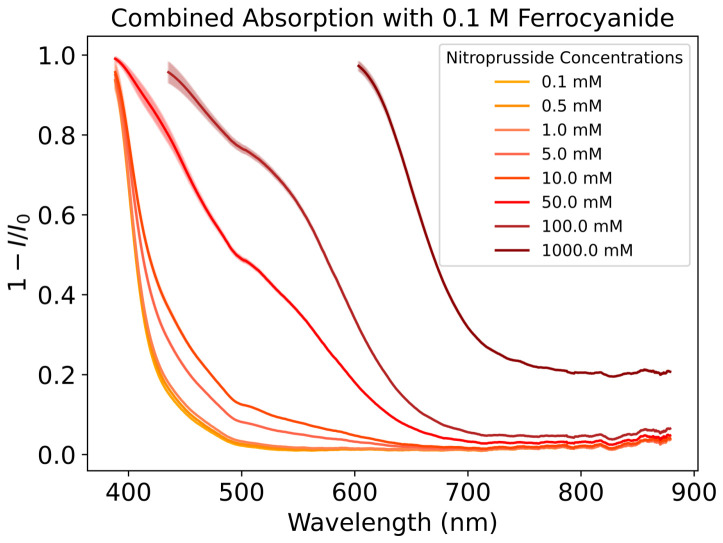
Relative absorption as a function of wavelength (nm). Combining mathematically the absorption curves of nitroprusside and ferrocyanide yields the following absorption curves. For all curves, a constant ferrocyanide concentration of 0.1 M was taken. This shows the distinct nitroprusside absorption feature appearing as a bulge in the spectrum.

We combined the absorption features of both ferrocyanide and nitroprusside to give the mathematical combination spectrum. We did this to later test the reliability of quantifying the mixture composition with FlareLab. Figure 7 shows the spectra resulting from the mathematical combination of the spectra from Figure 6 whilst keeping ferrocyanide at 0.1 M. It is evident that both species absorb in the same region; however, at higher concentrations, the presence of nitroprusside becomes noticeable as a bulge relative to the ferrocyanide feature (Figure 7). This is the 500 nm absorption peak, which is distinct from ferrocyanide absorbance and grows with concentration. At lower concentrations of nitroprusside, it is harder to distinguish between the different absorption curves at 500 nm; however, at higher concentrations, the correlation between nitroprusside concentrations becomes very clear. In this case, the ferrocyanide feature overlaps with the more distinguishable features of nitroprusside at lower concentrations. FlareLab does detect the effect of nitroprusside absorption at concentrations between 0.5 mM and 0.01 M as the spectrum does change with concentration, but it is difficult to accurately elucidate the concentration of nitroprusside in this range.

This principle might also be relevant in molecular mixtures with overlapping absorption characteristics, particularly when one species is detectable only through a feature that is less distinct at low concentrations.

### 3.3. Absorption Peaks

The various peaks of ferrocyanide and nitroprusside were tracked and are presented in Section 3.3.1 and Section 3.3.2. The mathematically computed peaks of the mixed standards are also compared to their experimental counterparts (Section 3.4).

#### 3.3.1. Ferrocyanide Peaks

**Figure 8:**
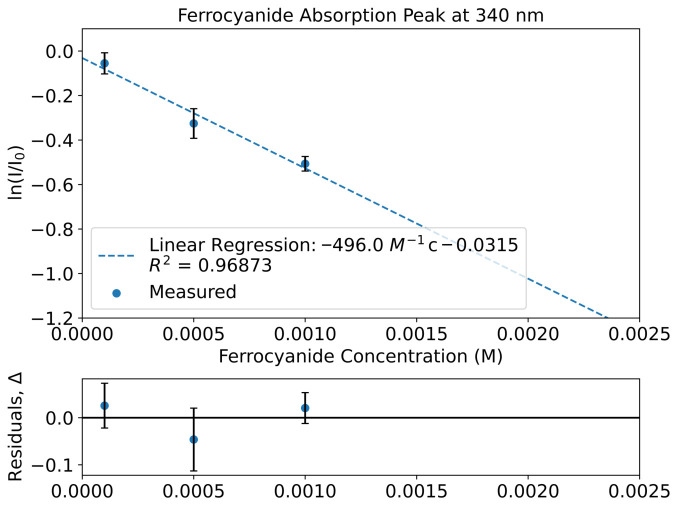
Evolution of ferrocyanide peaks at 340 nm depending on ferrocyanide concentration. Relative absorption, $\ln(I/I_{0})$, which is here derived from ln(1−r(λ)), is plotted as a function of ferrocyanide concentration (M). The plot follows the expected linear path.

Figure 8 shows the peak evolution of ferrocyanide. Ferrocyanide was found to have one peak in the UV–Vis region at 340 nm (Figure 6). Ferrocyanide at 340 nm shows a molar attenuation coefficient of $\varepsilon_{ferrocyanide}\left(340\;\mathrm{nm}\right)=\left(2.2\pm 0.4\right)\times 10^{3}\;{\mathrm{dm}}^{2}\;{\mathrm{mol}}^{-1}$.

#### 3.3.2. Nitroprusside Peaks

**Figure 9:**
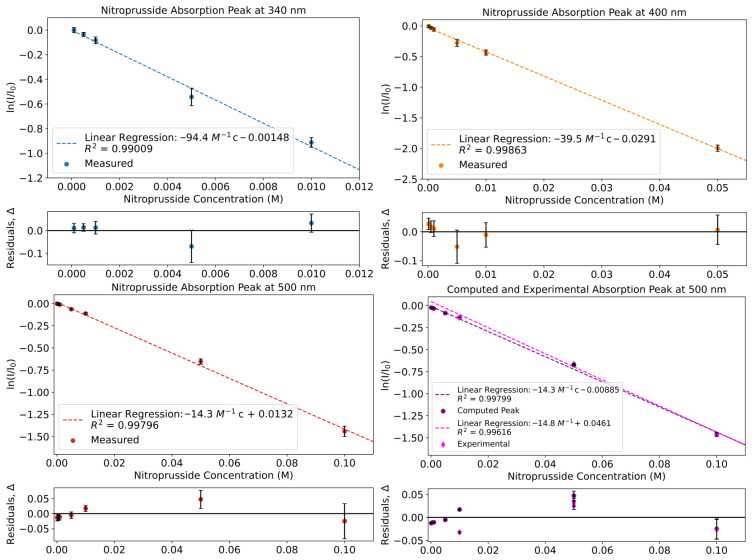
Relative absorption, $\ln(I/I_{0})$, which is here derived from $\ln(I/I_{0})=-\sigma n\beta$, is plotted as a function of nitroprusside concentration (M). We present the evolution of peaks of solutions containing nitroprusside only at 340 nm, 400 nm, and 500 nm, depending on the concentration of nitroprusside. The plot follows the expected linear path since $\ln(I/I_{0})=-\sigma n\beta$, where σ is the absorption cross-section, *n* the molecular number density and β the path length. The plot titled, “Computed and Experimental Absorption Peak at 500 nm”, contains a comparison between the computed value of the 500 nm peak and the experimental counterpart for nitroprusside. The mathematical combination containing a combination of the separately measured nitroprusside and ferrocyanide spectra (with ferrocyanide held at 0.1 M) is labelled in purple, and the experimental combination is labelled in magenta; these are plotted together. The experimental values line up well within the errors of the mathematical values. Both sets of values also line up well with the 500 nm peak points of the nitroprusside only solution; this was expected, as the 500 nm peak in the spectrum of nitroprusside plus ferrocyanide is attributed to nitroprusside solely. Both also follow the expected linear trend.

Nitroprusside shows 3 major features at 340 nm, 400 nm and 500 nm (Figure 6). By taking the results of the linear regression, we obtain a coefficient of $\varepsilon_{nitroprusside}\left(340\;\mathrm{nm}\right)=\left(4.1\pm 0.3\right)\times 10^{2}\;{\mathrm{dm}}^{2}\;{\mathrm{mol}}^{-1}$ for 340 nm. For the 400 nm peak, the coefficient was found to be $\varepsilon_{nitroprusside}\left(400\;\mathrm{nm}\right)=\left(1.71\pm 0.05\right)\times 10^{2}\;{\mathrm{dm}}^{2}\;{\mathrm{mol}}^{-1}$. For the 500 nm peak, the coefficient was $\varepsilon_{nitroprusside}\left(500\;\mathrm{nm}\right)=62.1\pm 1.7\;{\mathrm{dm}}^{2}\;{\mathrm{mol}}^{-1}$ (Figure 9).

If, instead, a 500 nm peak of the mathematical combination of ferrocyanide and nitroprusside was taken for the calculation, then the coefficient would be $\varepsilon_{nitroprusside}\left(500\;\mathrm{nm}\right)=62.0\pm 1.7\;{\mathrm{dm}}^{2}\;{\mathrm{mol}}^{-1}$; meanwhile, the experimental shows that $\varepsilon_{nitroprusside}\left(500\;\mathrm{nm}\right)=64.3\pm 1.2\;{\mathrm{dm}}^{2}\;{\mathrm{mol}}^{-1}$ (Figure 9). All three ε values for 500 nm are in the same region, which is expected since ferrocyanide does not absorb in this region significantly (Figure 6). Ferrocyanide is expected to have a negligible absorption cross-section at this wavelength range, and so nitroprusside is the primary contributor to ε in the combined cases.

The values between the 3 molar attenuation coefficients for 500 nm do differ slightly, but their error bars overlap. In combining ferrocyanide into the solution, it could be perturbing the solution and altering the value of ε slightly, and in the case of the mathematical combination the errors present in the ferrocyanide data have carried on in to the errors for the mathematical combination.

Nevertheless, when comparing the data from the mathematical combination and the experimental mixture at 500 nm, we clearly see that data points match up (Figure 9). This is encouraging as it shows that combining the standard data mathematically from separately measured standards matches the data from an equivalent experimentally made mixture.

Finally, all peaks are able to be fitted linearly. This is the expected Beer–Lambert progression, which shows that, whilst FlareLab has its limitations, FlareLab is able to reliably measure quantitative absorption curves.

### 3.4. Comparison of Mathematical and Experimental Combination

**Figure 10:**
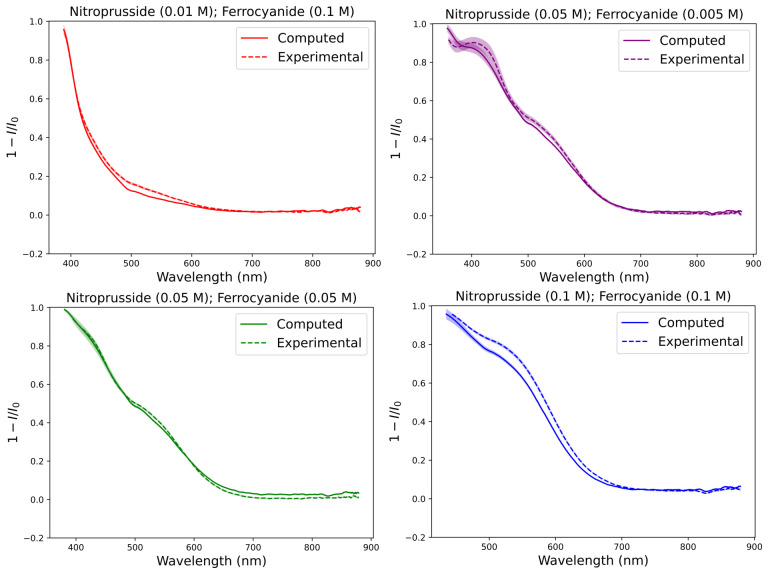
Relative absorbance as a function of wavelength (nm). The mathematical combination (solid lines) spectra line up with their experimental counterparts (dashed lines) for different combinations of ferrocyanide and nitroprusside concentrations. They show that the mathematical and experimental combinations line up well.

Comparing the full absorption spectra of the mathematical combination and their experimental counter parts shows that they largely match in terms of relative absorption where both nitroprusside and ferrocyanide have concentrations of 0.1 M (Figure 10). This could be due to increased scattering at higher concentrations of ferrocyanide and nitroprusside, causing the experimental combination to appear more absorbing than predicted by the sum of the individual attenuation coefficients. The deviations observed can inform us of the error inherent within our experimental setup when we use it for further experiments.

### 3.5. Lake Model Results

**Figure 11:**
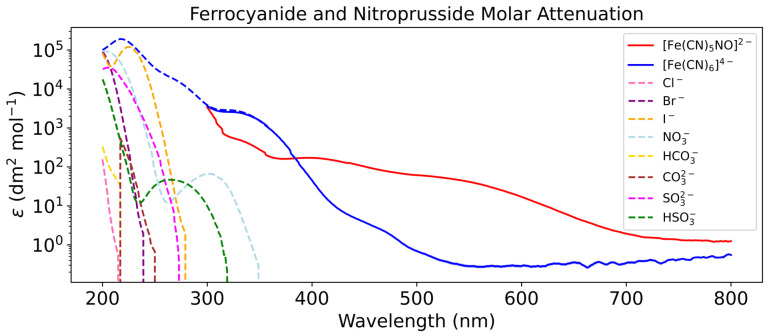
Molar attenuation coefficients of ferrocyanide (solid blue) and nitroprusside (solid red) are binned to 1 nm and are plotted with molar attenuation coefficients from Ranjan et al. Molar attenuation coefficients of ferrous, prebiotic lake compounds from Ranjan et al. are denoted using dashed lines [17]. Our coefficients were calculated by applying a linear regression to the log-scale molecular absorption (as a function of concentration) at each wavelength bin. We see good overlap between our data for ferrocyanide and that from Ranjan et al. For nitroprusside, we only have our data for λ≥300nm due to the limitations of our spectrometer. In the λ≥300nm region, compared to ferrocyanide, nitroprusside has a broader range where it absorbs light significantly. These wavelength-dependent coefficients (including data from Ranjan et al.) are fed into our lake model to attenuate the incoming solar flux as a function of molecular concentration, attenuation coefficient, and wavelength [17].

Given the absorptions shown in Figure 6, we can calculate a molar attenuation coefficients for each wavelength for ferrocyanide and nitroprusside (Figure 11). We measured the molar attenuation coefficients of ferrocyanide and nitroprusside corresponding to the wavelength region of 300–800 nm. As expected, in this region we see higher coefficients in a broader wavelength range for nitroprusside. However, we also included molar attenuation coefficients of ferrocyanide and other relevant ions in ferrous lakes as shown in Ranjan et al. [17]. We see good overlap between our data for ferrocyanide and that of Ranjan et al. [17].

We insert these coefficients into our lake model to determine $\chi^{n}\left(t\right)$, the time-dependent ratio of the UV flux integral between the layer *n* and the lake surface, (n=0). $k_{1}$ was attenuated using absorption data between 200 and 300 nm, whilst $k_{2}$ was attenuated using data between 300 and 400 nm, given that photo-oxidation of ferrocyanide occurs in the former region and photo-aquation in the latter region [33]. This was performed for total depths of 1 m and 5 m.

#### 3.5.1. One-Meter Lake

**Figure 12:**
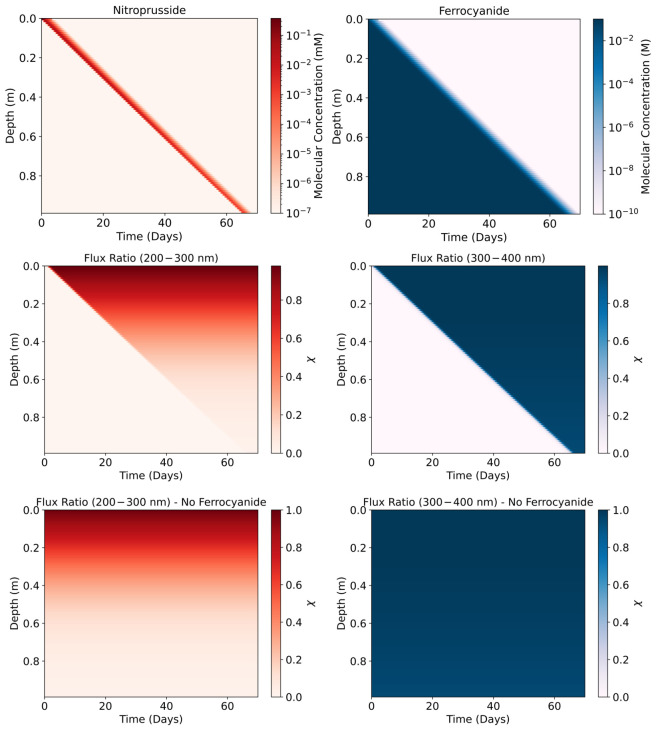
Lake model results, showing how long ferrocyanide survives (days) at different lake depths (m), with a depth of 1 m. We also show how the rate constants $k_{1}$ (200–300 nm) and $k_{2}$ (300–400 nm) are attenuated by plotting how the attenuation factor χ changes with depth and time. This was performed with and without ferrocyanide in the model, with the latter variant labelled with ‘no Ferrocyanide’. As UV light degrades the upper layers, destroying the ferrocyanide that attenuates UV flux, the lower layers are exposed. Thus, the lower-level ferrocyanide starts to degrade gradually exposing the even deeper layers to UV flux. Therefore, deep ferrocyanide survives longer than surface-level ferrocyanide. Nitroprusside can only be produced in the layers that are exposed to UV flux. Simultaneously, nitroprusside is photochemically degraded. With time, a nitroprusside layer travels down the lake.

Using the attenuated rate constants, we constrain the time-dependent concentrations of ferrocyanide and nitroprusside at different depths of the lake (Figure 12). Without a source of more ferrocyanide, it decays at different rates, depending on the lake depth. Ferrocyanide survives longer at lower depths, as the upper layers effectively block incoming UV reaching lower in the lake. With time, ferrocyanide decays in the upper layers, allowing for greater UV flux to reach deeper, exposing the lower layers. This process continues until all ferrocyanide decays. As UV flux travels down the lake, a layer of nitroprusside is formed from ferrocyanide. It does not survive long as UV light also destroys nitroprusside. The nitroprusside therefore travels down the lake column as successive layers are exposed to UV. Ferrocyanide therefore provides the lake with a UV shadow and the resulting nitroprusside, owing to its own ability to attenuate UV light, acts as the shadow of the shadow, further protecting the deeper layers.

We also see that the maximal nitroprusside concentration that is produced in each layer decreases with depth. This is due to the absorbers—according to Ranjan et al., they are mainly active in the 200–300 nm range, which hinders nitroprusside production, but they are not active in the 300–400 nm range, which is responsible for nitroprusside destruction [17]. This is further supported when we run our model with no ferrocyanide. We see that the extra absorbers attenuate $k_{1}$ but not $k_{2}$ (Figure 12). If instead we remove the absorbers from Ranjan et al. and run the model with just ferrocyanide, we find that the life time of ferrocyanide is unchanged and that the same maximal concentration of $10^{-1}$ mM of nitroprusside is eventually reached at each layer [17].

**Figure 13:**
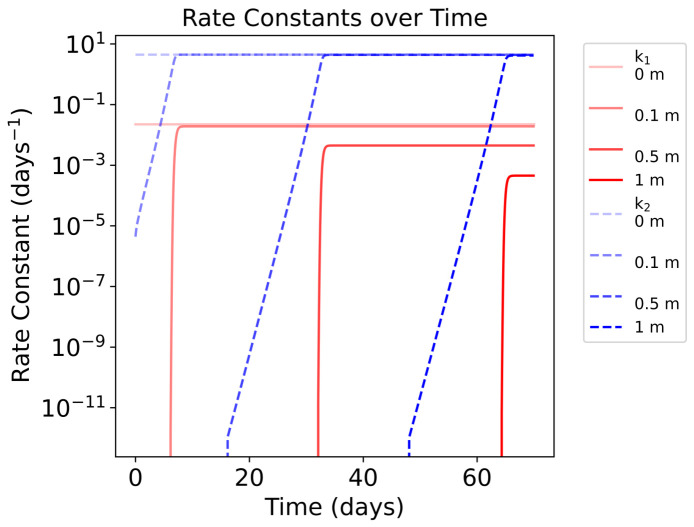
Change in rate constants (days^−1^) as a function of time (days) for different depths, with a total depth of 1 m. The dashed blue lines represent $k_{2}$ values, i.e., the rate constant for photo-aquation of ferrocyanide and nitroprusside; the solid red lines represent the $k_{1}$ values, i.e., the rate constant for photo-oxidation of ferrocyanide. As UV penetrates deeper in the lake with time, the rate constants at successive layers increase in value.

Once a layer is exposed, the production of nitroprusside and decay of ferrocyanide proceeds rapidly. This is evidenced by the rapid increase in the rate constants’ values as UV light reaches a layer (Figure 13). The rapid increase in $k_{1}$ and $k_{2}$ values explains the low survivability of both compounds once exposed to UV. Whilst the absorptive properties of ferrocyanide and nitroprusside demonstrate good UV attenuation, significantly protecting lower layers from photochemical reactions, acting as sunscreens, the system cannot be maintained for long without a sustained, good source of ferrocyanide. These results show a plausible upper limit of the abundances of ferrocyanide and the resulting abundance of nitroprusside. Under these favourable conditions, we can expect deeper lake layers to remain protected for a time period in the order of months. Higher depths than shown in the model could remain protected for longer, potentially allowing for some more complex compounds to form without harm from UV light. However, we do not consider convection or diffusion within the lake, nor do we consider thermal degradation rates of ferrocyanide or nitroprusside. These factors may decrease the time that deeper layers remain protected.

#### 3.5.2. Five-Meter Lake

**Figure 14:**
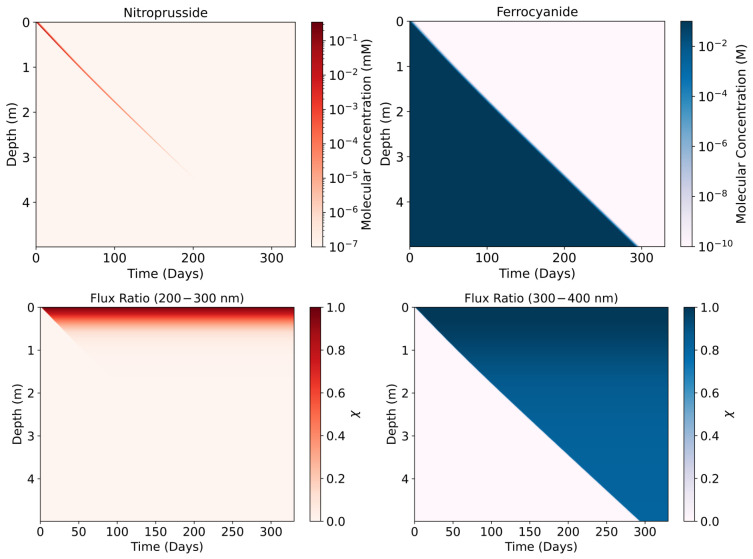
Lake model results, showing how long ferrocyanide survives (days) at different lake depths (m), with a total depth of 5 m. We also show how the rate constants $k_{1}$ (200–300 nm) and $k_{2}$ (300–400 nm) are attenuated by plotting how the attenuation factor χ changes with depth and time. As UV light degrades the upper layers, destroying the ferrocyanide that attenuates UV flux, lower layers are exposed. Thus, lower level ferrocyanide starts to degrade gradually exposing the even deeper layers to UV flux. Therefore, deep ferrocyanide survives longer than surface-level ferrocyanide. Nitroprusside can only be produced in the layers that are exposed to UV flux. Simultaneously, nitroprusside is photochemically degraded. With time, a nitroprusside layer travels down the lake.

**Figure 15:**
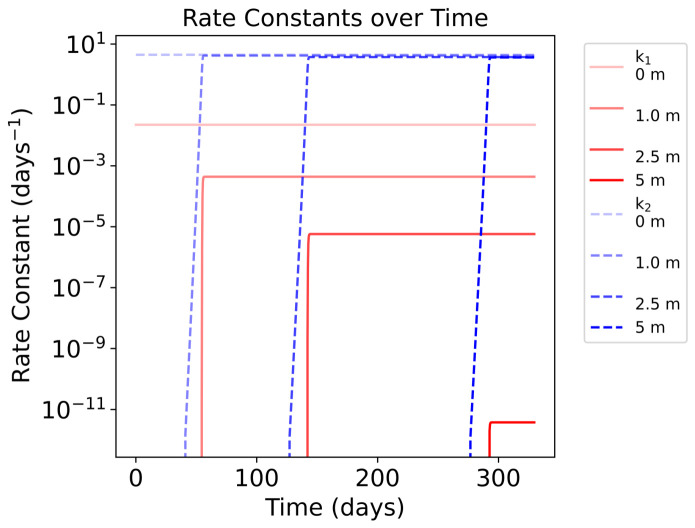
Change in rate constants (days^−1^) as a function of time (days) for different depths, with a depth of 5 m. The dashed blue lines represent $k_{2}$ values, i.e., the rate constant for photo-aquation of ferrocyanide and nitroprusside; the solid red lines represent the $k_{1}$ values, i.e., the rate constant for photo-oxidation of ferrocyanide. As UV penetrates deeper in the lake with time, the rate constants at successive layers increase in value.

Similar to the 1 m lake results, we see the nitroprusside formed and destroyed with each exposed layer, whereas ferrocyanide sequentially degrades with each layer (Figure 14). Due to the presence of UV absorbers, as shown by Ranjan et al., we see that $k_{1}$ is rapidly attenuated with depth [17]. Simultaneously, the absorbers are also slightly active in the 300–400 nm region, so we can see a slight depth-dependent attenuation of $k_{2}$ as a result. However, it is clear that this effect is minimal and the dominant UV attenuators in this region are ferrocyanide and nitroprusside (Figure 15).

#### 3.5.3. Ferrocyanide Shadow

**Figure 16:**
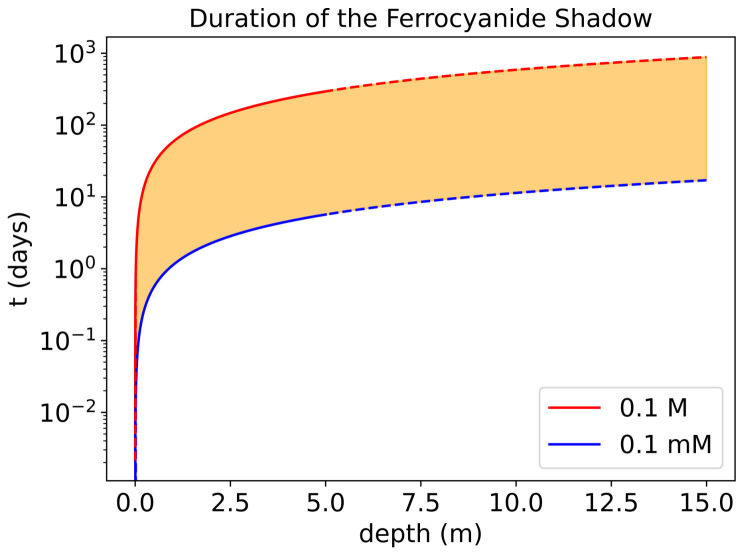
Duration of the ferrocyanide shadow. The y axis shows the total amount of time ferrocyanide survives depending on lake depth in log scale. Total survival depends on starting ferrocyanide concentration, with red lines starting with 0.1 M of ferrocyanide and blue lines with 0.1 mM of ferrocyanide. Solid lines are total survival times taken from our lake model (5 m total depth) and dashed lines are extrapolations to greater lake depths. The extrapolation results from a linear regression based on our model results. The shaded region therefore represents the possible total survival times of ferrocyanide when a lake starts with ferrocyanide concentrations between 0.1 mM and 0.1 M.

Since we were interested in calculating the upper limit of ferrocyanide survival to gauge its potential as a sunscreen, we chose favourable conditions and a very high initial ferrocyanide concentration of 0.1 M, noting that the solubility of ferrocyanide would limit higher concentrations. However, other works assumed concentrations of 0.1 mM [17,44]. We ran our model with both initial concentrations for a 5 m deep lake. For both 1 m and 5 m lakes we notice that the total survival time of ferrocyanide largely grows linearly with depth (Figure 12 and Figure 14). Examining this slope, we see that total survival time $t=1.14\;m^{-1}\times d$, where d is the depth, for a lake with an initial concentration of 0.1 mM. For an initial concentration of 0.1 M, the total survival time $t=58.9\;m^{-1}\times d$. Using these relationships, we extrapolate the total survival time of ferrocyanide to deeper lake depths. At 5 m, ferrocyanide survives on the order of days if the initial concentration is 0.1 mM, while this survival time increases to on the order of months with an initial concentration of 0.1 M. Any lake with ferrocyanide concentrations in the range of 0.1 mM–0.1 M would show survival times from days up to years, highlighting the strong dependence of ferrocyanide survival on both initial concentration and lake depth (Figure 16).

## 4. Discussion

The results show that ferrocyanide and nitroprusside can act as effective sunscreens. Absorptions show that UV vulnerable molecules that lie deeper in a lake could be protected for a time on the order of days to months. Irradiation at 260 nm, which is responsible for nucleotide chain degradation, could be significantly blocked by meaningful concentrations of ferrocyanide and nitroprusside [17]. Ferrocyanide was found to have the dominant impact, due to the higher concentrations of ferrocyanide that were present longer at various lake depths compared to nitroprusside (Figure 12). This was true, given the favourable conditions that are also conducive to efficient ferrocyanide production [33]. Under less favourable conditions, we expect ferrocyanide to have a lower impact on UV flux attenuation.

Without a source of ferrocyanide, it will decay in a rapid geological timescale, leaving lake layers unprotected. A source of ferrocyanide could be excess ferrocyanide that was deposited on the lake floor (see Section 2.4.1). Exchange of ferrocyanide between solid and aqueous phases could allow for a flux of aqueous ferrocyanide to exist beyond the post-impact HCN rainout. This could extend the sunscreen effect ferrocyanide provides to the lake; however, more work is needed to determine the impact equilibria between phases have on this system.

Other ways of allowing ferrocyanide survive longer were explored by considering self-shielding and shielding effects [33,59]. Self-shielding was used to explain why ferrocyanide at higher concentrations survived longer to UV irradiation [33]. We see evidence of this, as our lake model shows deeper layers being shielded from ferrocyanide in upper layers. Once a layer is exposed, the rate of ferrocyanide decay increases rapidly. A high concentration of ferrocyanide within a layer mutually protects the complexes from UV photons, but as some react, this effect weakens and starts a positive feedback that accelerates the rate of reaction. Todd et al. cites the effect nucleosides have on increasing the survivability of 2-animoxazole, which are susceptible to photochemistry at a wavelength of 200 nm [59]. The production of nitroprusside is mostly responsive to wavelengths between 200 and 300 nm, which indicates that these nucleosides could also slow the rate of reaction to form nitroprusside, increasing the survival of ferrocyanide. Ranjan et al. explores UV transmission in natural waters [17]. Ions like Br^−^, ${\mathrm{Fe}}^{2+}$, and I^−^ could further attenuate UV irradiation between 200 and 250 nm in prebiotic ocean waters, whilst ${\mathrm{SO}}_{3}^{2-}$, in addition to the latter two ions, could do the same in prebiotic freshwater environments at the wavelengths 200–280 nm [17,58]. Because the molar attenuation coefficients of these ions are generally significantly lower in the UV region than ferrocyanide and nitroprusside, their concentrations would have to be large to meaningfully contribute to UV attenuation [17]. The same applies to photoproducts like ferricyanide and cyanate [60,61]. Assuming this is possible, the production of nitroprusside could be further attenuated allowing ferrocyanide to survive longer. However, effects that only attenuate UV irradiation at 200–300 nm do not attenuate the photo-aquation of ferrocyanide, which mainly occurs at 300–400 nm [33]. Without absorbers within the photo-aquation range or a significant sustained flux of ferrocyanide, the effects described above are likely to only have a marginal impact on the survival of ferrocyanide and the system at large.

We tested our model without the extra absorbers from Ranjan et al. and ran the model with just ferrocyanide, and found that the life time of ferrocyanide is unchanged and that the same maximal concentration of $10^{-1}$ mM of nitroprusside is eventually reached at each layer [17]. Furthermore, the extinction coefficients of pure water dominate over its scattering coefficients, meaning that at the length scales of our model, UV scattering is negligible and the absorbances of UV due to the dissolved compounds are the main factors [17]. This proves the aforementioned point, that in order to extend the protection that ferrocyanide provides, it must itself be protected by an absorber in the 300–400 nm region. Of course, the life time of ferrocyanide, and subsequently the production of nitroprusside, is heavily dependent on the amount of ferrocyanide available initially. Other works assume a 0.1 mM concentration of ferrocyanide [17,44]. If we use this concentration, we see that the life time of ferrocyanide decreases to less than 5 days (at a depth of 5 m). This is in line with the conclusions presented by Todd et al., which state that ferrocyanide survival is prolonged by higher initial concentrations [33]. As we are interested in the upper limits of the timescales involved with ferrocyanide and other photosensitive compounds, any realistic lakes with ferrocyanide concentrations below 0.1 M will naturally present survival rates below our upper limit (Figure 16).

At this stage, it is important to acknowledge the properties of other known sunscreens, that could have been present in primordial environments, and how they compare to ferrocyanide and nitroprusside. Mycosporines show large absorptions in the 300–400 nm region, which makes them suitable UV blockers; however, they are often associated with more complex biochemical systems like cyanobacteria. For this reason, ferrocyanide and nitroprusside are more abiotically accessible in the ferrous lake [62]. Avobenzone shows good UV absorption in the 300–400 nm initially; however, with UV irradiation this absorption shifts to the 200–300 nm due to a breaking of the O-H-O bond across the two central carbonyls [63]. ZnO absorbs well in the high energy UV region, yet is fluorescent in the 300–400 nm region [64]. ZnO and avobenzone both would poorly protect the 300–400 nm, leaving compounds like NADH exposed to UV irradiation. Ferrocyanide and nitroprusside therefore represent more abiotically accessible and photochemically stable sunscreens in the 300–400 nm region.

Ferrocyanide, if supported by other absorbers, could survive for a significant period under favourable conditions. This would also be true if there was a significant flux of ferrocyanide, matching or exceeding the rate of ferrocyanide destruction, thus replenishing destroyed ferrocyanide. This would allow it to protect compounds deeper in the lake which are vulnerable to UV irradiation for significant periods. Nucleotide chains, susceptible to degradation at 260 nm, would survive and be allowed to form polynucleotide chains [17,18]. Nitroprusside would form and degrade mainly at the surface of the lake. Thus, methyl isocyanide would also form on the surface of the lake. With stagnant conditions, it would have to slowly diffuse to the lower layers where it could abiotically link nucleotides and amino acids [9]. Especially if a constant source of ferrocyanide existed, the production of nitroprusside could continue, by extension acting as a source of methyl isocyanide at the surface of the lake. Methyl isocyanide would continuously diffuse to the lower layers where polynucleotide and polypeptide chains could consistently be made, protected from UV degradation.

Largely linear relationships between peak size and concentration of nitroprusside and ferrocyanide are observed. The linear combinations of the two standards line up with the corresponding experimental mixtures, which increases our confidence in the measured absorptions. It is therefore possible to calculate the molar attenuation coefficients from these relationships using FlareLab, and feed these values into our lake model to estimate the upper limit of ferrocyanide and nitroprusside survival.

**Figure 17:**
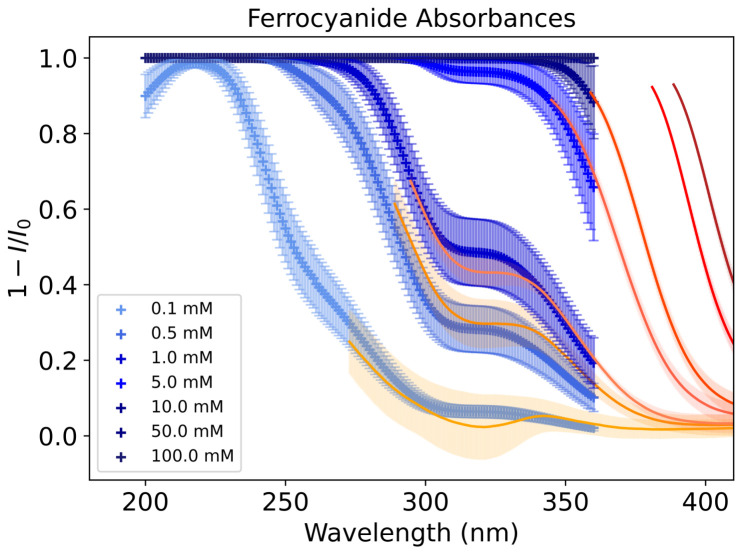
Comparison of our ferrocyanide spectra with spectra derived from the molar attenuation coefficients from Ranjan et al. [17]. Data from Ranjan et al. was converted into expected relative absorbance, dependent on concentration of ferrocyanide, and plotted against our data [17]. The blue scatter plots show the ferrocyanide absorbances based on data from Ranjan et al., whereas the solid line plots show our data and their colours have the same meanings as those in Figure 6 [17].

We compare our data to other available UV–Vis data to further validate our results. Rucki shows the spectrum of nitroprusside in the visible region; we can confirm their observed peaks at 400 nm and 500 nm with our results [43]. By comparing our ferrocyanide absorptions with those that we would expect from using the molar attenuation coefficients from Ranjan et al., we see overlap [17]. The higher concentrations do not overlap as data from Ranjan et al. does not include the wavelengths at which we report our absorptions, nevertheless, it confirms our observations of large regions of featureless ferrocyanide absorption (Figure 17) [17]. For these reasons, we are confident in our measured features of nitroprusside and ferrocyanide.

FlareLab has limitations. We optimise our exposure time to minimise our noise; however, at lower wavelengths (<300 nm), the UV–Vis FLAME-S-UV-Vis Spectrometer measures a large amount of noise. Because of the performance of our spectrometer, the noise baseline is increased when concentrations are low and more light is let through the sample. For higher concentrations noise dominates in regions where light intensity is low due to heavy absorption by the sample. Background features are recovered in these areas where the detector becomes unable to distinguish between peaks resulting from any differences in absorption. This leads to regions of unreliable data, as these measured features are not solely due to the absorption of light by the compounds. Because this effect depends on concentration and compound, it limits the measurement range of FlareLab, depending on those two factors. We show that ferrocyanide and nitroprusside absorption is linear with respect to the logarithm of the compounds’ concentrations and that the shown broader absorption features are accurate, but beyond this range, we cannot say if our measurements are reliable (Figure 6 and Figure 7).

This also limits our ability to calculate molar absorption coefficients below 300 nm and therefore limits the effectiveness of our lake model. In truth, photo-oxidation of ferrocyanide occurs at wavelengths below 300 nm and photo-aquation above 300 nm [33]. We were able to add absorbance data for ferrocyanide in the 200–300 nm, as well as other typical absorbers in ferrous lakes, but have no such data for nitroprusside [17]. This means that we were able to run our model for the 200–800 nm region, with separate attenuation for $k_{1}$ and $k_{2}$, i.e., the rate constants for photo-oxidation and photo-aquation, respectively, albeit with incomplete nitroprusside data. We only have molar attenuation coefficients values of nitroprusside from 300 to 800 nm, we therefore can only calculate its UV attenuation contribution from 300 nm in our model. Given Figure 11, we can see that for low wavelengths both compounds absorb strongly. Therefore, we expect that the molar attenuation coefficients of nitroprusside below 300 nm to be similarly high (or higher). This would indicate that photo-oxidation rates would be strongly attenuated by the presence of nitroprusside or ferrocyanide and in a ferrocyanide rich lake only the exposed lake layer would photo-oxidise to nitroprusside, just as our current model predicts. This further reinforces our conclusion that ferrocyanide and nitroprusside are good sunscreens. Measurements of ferrocyanide absorptions have been conducted by others; however, here we present a more extensive study of the absorptions of both ferrocyanide and nitroprusside [17,33,59]. To build a more comprehensive picture of the absorptive properties of prebiotic compounds, measurements of nitroprusside absorption between 200 and 300 nm could be undertaken in future.

We considered a completely stagnant lake in a post-impact climate, analogous to the lakes presented in Walton et al., whilst ignoring the thermal stabilities of nitroprusside and ferrocyanide [30]. However, even in a completely stagnant lake, the internal diffusion of water would mix the layers in a timescale of roughly 31 years [65]. This means that, lower-lying material may end up diffusing to the surface with time and would end up becoming exposed to UV light in the upper layers. Given that the timescale of diffusion is much slower than our timescale of ferrocyanide decay, we did not need to consider this effect in the model. Nevertheless, if ferrocyanide existed in an environment that allowed it to survive for timescales on the order of tens of years or longer, we need to take diffusion into account. If mixing in the lake was significant, then our model would not apply the same way. Diffusion and currents would explicitly need to be accounted for to determine the effectiveness of the sunscreens and the timescales that vulnerable products would realistically spend exposed to UV light. Generally, we would expect the effectiveness of the sunscreens to be diminished by the degree of mixing in the lake.

Given the self-similarity in the results from Todd et al. with respect to lake area, we expect the production of nitroprusside to scale with differing lake areas [33]. Our overall conclusion, that ferrocyanide and nitroprusside act as good sunscreens, should not be affected. The limit to this reasoning is if we consider very large bodies of water, eventually the size will be too large for meaningful concentrations to build up.

Whilst our model is informative, establishing the effectiveness of organometallic iron–cyanides as sunscreens, it would be improved by considering a wider array of reactions, for example the photo-aquation of ferrocyanide is reversible, yet the reversibility was not considered in the present model. One such way to regenerate ferrocyanide involves the use of sulphite, which could come from surface hydrothermal vents [10]. It would also be important to investigate scenarios that involve less ideal conditions, such as pH = 7 or T = 300 K, as these lower the survivability of ferrocyanide significantly [33]. Thermal degradation of ferrocyanide and nitroprusside should also be incorporated for a more complete picture. Diffusion and currents in the lake would perturb the layers within the lake and drag otherwise protected compounds to the surface, exposing them to UV and potentially degrading them. It would be of great interest to understand how much perturbation the layers can withstand before diffusion must be considered. Such factors (further and alternative reactions, thermal stability, and pH) could significantly alter the lifetimes of nitroprusside and ferrocyanide. For example, an acidic lake at high temperatures would lower the lifetime of ferrocyanide and likely nitroprusside too [33]. Under those conditions, these compounds could not act as effective sunscreens. By contrast we considered a high pH, low temperature lake to maximise the survival of these compounds with respect to these factors. We believe that further considering these factors are valuable next steps to build a more comprehensive picture concerning how prebiotic environments could be constructed within the context of the cyanosulphidic network. The effect of shielding that ferrocyanide provides to lake environments could potentially contribute an answer to the tension that the cyanosulphidic network has, provided that ferrocyanide can be maintained within the lake for significant periods of time, which is that UV is needed to construct the base material; simultaneously, more complex compounds are also susceptible to UV degradation [46].

Examples of biomolecules that ferrocyanide and nitroprusside could protect are adenosine triphosphate, ATP, and nicotinamide adenine dinucleotide, NADH; both are central to cellular metabolism [66,67]. ATP is considered the energy currency of the cell, whilst NADH allows for the regeneration of ATP via oxidative phosphorylation [67]. Both could have initially been formed abiotically; however, this is still strongly debated [67]. Precursors for ATP have been found on meteorites [67,68,69]. Different formation pathways of ATP have been proposed [70,71,72,73]. Often, this requires the presence of ribose, adenine, and large quantities of HCN [67]. HCN could have persisted on the early Earth via multiple pathways [47,74,75,76,77,78,79]. Meanwhile, NADH precursors have also been identified on several meteorites and interstellar cometary dust [80,81,82,83]. NADH components could have been synthesised abiotically under early Earth conditions, whilst several pathways have been proposed to form NADH itself or its deprotonated form, NAD^+^ [67,84,85,86,87,88,89].

Both ATP and NADH are susceptible to UV degradation. ATP is susceptible to UV radiation at around 260 nm, akin to polynucleotides (such as DNA and RNA chains) [17,18,26]. Meanwhile, NADH is susceptible to radiation in the 300–400 nm region [27]. Scaling the half-life of NADH with the actinic surface flux we use, we find that the half-life would have been 6 months on the surface of the lake [27]. ATP would have been protected well by both the salts from Ranjan et al., ferrocyanide, and nitroprusside; assuming the salts do not degrade, this protection could last indefinitely [17].

Experimental works confirm the protective properties of the salts from Ranjan et al. [17,44]. The survival of glycine in carbonate ponds (containing non-ferrous salts from Ranjan et al.) and ferrocyanide ponds was investigated [17,44]. It was found that glycine is better protected under >300 nm irradiation by carbonate ponds, due to the eventual degradation of ferrocyanide, which also leeches iron from the system due to the formation of pyrite and goethite becoming more favourable as a result. This is not an issue for carbonate ponds [44]. However, salts in carbonate ponds primarily protect compounds from irradiation below 300 nm. Furthermore, iron fluxes from streams, as reported by Todd et al. and Toner et al., could compensate for iron leaching, reducing the distinction between carbonate and ferrocyanide ponds in this wavelength region [33,35].

**Figure 18:**
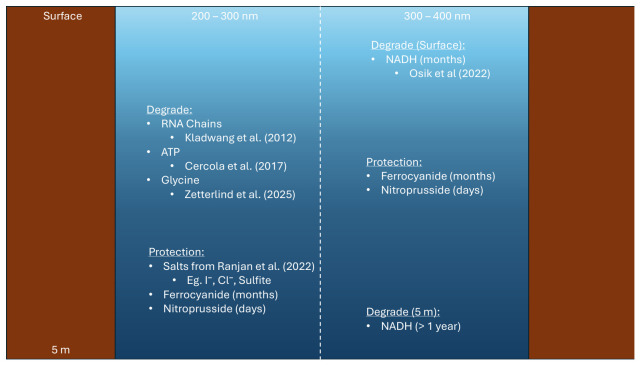
Compounds protected from UV degradation in the regions of 200–300 nm and 300–400 nm, and which compounds are susceptible to such irradiation. Ferrocyanide, nitroprusside, and typical salts found in ferrous lakes all protect compounds susceptible to 200–300 nm, e.g., polynucleotide chains, ATP, and glycine [17,18,26,44]. If salts like I^−^, Cl^−^, and ${\mathrm{SO}}_{3}^{2-}$ can remain, such protection could be indefinite. Ferrocyanide and nitroprusside protects compounds such as NADH in the 300–400 nm region [27]. In the absence of ferrocyanide regeneration and ferrocyanide fluxes, this protection could change the half-life of NADH from 6 months at the surface to two months more at a depth of 1 m and beyond 1 year at a depth of 5 m.

Ferrocyanide and nitroprusside are able to absorb UV irradiation and thus offer protection to compounds above 300 nm. Therefore, compounds such as NADH would only be protected by ferrocyanide and nitroprusside. In the absence of ferrocyanide regeneration or fluxes, this would allow for NADH to survive for two extra months at a depth of 1 m (owing to the fact that this is the time needed for 1 m layer to become exposed to UV irradiation), whereas at a depth of 5 m NADH survival could extend beyond 1 year in the lake. It is possible that precursor molecules with similar structure would also rely on the protection provided by the transition metal complexes (Figure 18). Furthermore, whilst we use NADH as a case study, any molecule susceptible to 300–400 nm would benefit from the protection provided by ferrocyanide and nitroprusside, and have their lifetimes similarly extended.

Finally, we would like to note that whilst we have focused on how UV shielding could benefit the cyanosulphidic scenario, by allowing the safe build-up of more complex compounds due to the presence of ferrocyanide and nitroprusside, the persistent absence of UV could also inhibit the progression of the cyanosulphidic network [46]. Selection pressure that UV could have provided towards the canonical bases, would also not occur via this mechanism in deeper lakes if ferrocyanide is regenerated [21,90]. Consequently, lakes with a sustained presence would have the majority of the chemistry happen at the surface, which would then have to diffuse downwards to avoid destruction [17].

## 5. Conclusions

We characterised the absorptions of ferrocyanide and nitroprusside and built a toy lake model to estimate the survival of ferrocyanide and the production of nitroprusside. We were able to accurately characterise the absorptions of ferrocyanide and nitroprusside, as well as their molar attenuation coefficients. We found that in the absence of ferrocyanide sources, the survival of ferrocyanide in the lowest layers of a 1 m lake is on the order of months and nearly a year in lakes with a total depth of 5 m, due to significant UV attenuation. Other lower-lying UV sensitive compounds would be significantly protected. A nitroprusside layer forms in the exposed layers of the lake and travels down as UV degrades both ferrocyanide and nitroprusside. With the presence of compounds that further attenuate UV flux, as well as accounting for the reversibility of photo-aquation, the survival of ferrocyanide could potentially be extended significantly [10,17,33]. Therefore, ferrocyanide and nitroprusside could act as good sunscreens, with nitroprusside acting as the shadow of the shadow, to protect compounds at greater lake depths. The protection afforded by ferrocyanide and nitroprusside is especially necessary to protect NADH, as other typical ferrous lake salts do not absorb in the 300–400 nm region where NADH is susceptible [17,27]. The presence of both complexes would therefore allow for the key compounds used in cellular metabolism to build up in prebiotic lakes. For the resultant lake chemistry to be accessible and for the sunscreen effect to be sustainable in the long-term, such lakes would likely require in-flows of Fe(II) and outflows to facilitate the spread of more complex products. We present a comprehensive study of ferrocyanide and nitroprusside absorption across the UV–Vis spectrum. By incorporating previous absorption data, we were able to build a model across a broad spectral range that constrains the survival of compounds of interest in the origins community such as NADH, ATP, and polynucleotide chains as well as the survival of ferrocyanide and nitroprusside itself. The stellar spectrum used to scale the rate constants (in our case the early Sun) can be changed in our model, which would allow for similar investigations of survival to be completed for exoplanets around different stars. Akin to the abiogenesis zone discussed in Rimmer et al., our model could be used to build similar zones around different stars of interest [1]. Combined, this work details the UV environment shallow stagnant lakes—thought to be key in the cyanosulphidic scenario—have with the presence of ferrocyanide and nitroprusside, and contributes to the discussion surrounding the build up of biochemical stock in the context of the early Earth as well as to the discussion surrounding the prevalence of the origin of life in our Universe [17,30,33]. 

## Data Availability

All data is available at https://dataverse.harvard.edu/dataset.xhtml?persistentId=doi:10.7910/DVN/QAZ9B6 (accessed on 23 May 2023).

## Appendix A. Tables

**Table A1:** Table for the nitroprusside standards. The initial concentrations, $c_{i}$, are the concentrated solutions that are diluted to the desired concentrations. α is the dilution factor. The final concentrations, $c_{f}$, are the sample concentrations for which spectra are measured, where $c_{f}=\alpha \cdot c_{i}$. The total cuvette volume for each sample is always 2 mL, but the volume of the concentrate solution (volume of $c_{i}$) is shown in the square brackets. Unless otherwise stated, all volumes are shown in units of mL.

Initial Concentration	Final Concentration	Dilution Factor	Cuvette Volume
$c_{i}$	$c_{f}$	α	$V_{c}$
(M)	(M)		(mL)
1	1	1	2 [2]
1	$10^{-1}$	1/10	2 [0.2]
1	$5\times 10^{-2}$	1/20	2 [0.1]
1	$10^{-2}$	1/100	2 [0.02]
1	$5\times 10^{-3}$	1/200	2 [0.01]
1	$10^{-3}$	1/1000	2 [2 μL]
1	$5\times 10^{-4}$	1/2000	2 [1 μL]
0.001	$10^{-4}$	1/10	2 [200 μL]
0.001	$5\times 10^{-5}$	1/20	2 [100 μL]
0.001	$10^{-5}$	1/100	2 [20 μL]
0.001	$5\times 10^{-6}$	1/200	2 [10 μL]
0.001	$10^{-6}$	1/1000	2 [2 μL]

**Table A2:** Table for the ferrocyanide standards. The initial concentrations, $c_{i}$, are the concentrated solutions that are diluted to the desired concentrations. α is the dilution factor. The final concentrations, $c_{f}$, are the sample concentrations for which spectra are measured, where $c_{f}=\alpha \cdot c_{i}$. The total cuvette volume for each sample is always 2 mL, but the volume of the concentrate solution (volume of $c_{i}$) is shown in the square brackets. Unless otherwise stated, all volumes are shown in units of mL.

Initial Concentration	Final Concentration	Dilution Factor	Cuvette Volume
$c_{i}$	$c_{f}$	α	$V_{c}$
(M)	(M)		(mL)
0.1	$10^{-1}$	1	2 [2]
0.1	$5\times 10^{-2}$	1/2	2 [1]
0.1	$10^{-2}$	1/10	2 [0.2]
0.1	$5\times 10^{-3}$	1/20	2 [0.1]
0.1	$10^{-3}$	1/100	2 [20 μL]
0.1	$5\times 10^{-4}$	1/200	2 [10 μL]
0.1	$10^{-4}$	1/1000	2 [2 μL]
0.001	$5\times 10^{-5}$	1/20	2 [100 μL]
0.001	$10^{-5}$	1/100	2 [20 μL]
0.001	$5\times 10^{-6}$	1/200	2 [10 μL]
0.001	$10^{-6}$	1/1000	2 [2 μL]

**Table A3:** Concentrations of the experimental mixed standards. Note: “( )” show the masses, if relevant, added to the sample to make the desired concentration. In the case of nitroprusside, sodium nitroprusside dihydrate was used; “[ ]” show the volume of the concentrate solution used to dilute to the desired concentration. For nitroprusside, a concentrate solution of 1 M was used. For ferrocyanide, a concentrate solution of 0.1 M was used.

Nitroprusside	Ferrocyanide
0.1 M	0.1 M
(59.6 mg)	[2 mL]
0.01 M	0.1 M
(6.0 mg)	[2 mL]
0.05 M	0.05 M
[0.01mL]	[1mL]
0.05 M	0.005 M
[0.1mL]	[0.01mL]
